# Comparison of the analgesic effect of dezocine and esketamine in combination with sufentanil respectively after laparoscopic cholecystectomy: a prospective randomized controlled study

**DOI:** 10.1186/s12871-024-02430-y

**Published:** 2024-02-05

**Authors:** Boran Deng, Dingding Wang, Zifeng Xie, Yongqin Wang, Li Huang, Manlin Jiang, Tu Shen

**Affiliations:** 1 Department of Anesthesiology, The First Affiliated Hospital of Jinzhou Medical University, Jinzhou City, 121000 China; 2 Department of Anesthesiology, Eye &ENT Hospital of Fudan University, Shanghai City, 200031 China; 3 First Clinical Medical College, Jinzhou Medical University, Jinzhou City, 121000 China; 4 Anesthesiology Surgery Center of Zigong Hospital of Traditional Chinese Medicine, Zigong City, 643000 China

**Keywords:** Laparoscopic cholecystectomy, Esketamine, Dezocine, Analgesic effect, Inflammatory factors

## Abstract

**Background:**

Sufentanil in combination with dezocine or esketamine is often used for postoperative analgesia. However, there is a lack of clinical evidence of efficacy. This study compares the analgesic effects of esketamine and dezocine combined with sufentanil for relieving pain after laparoscopic cholecystectomy(LC).

**Methods:**

A total of 58 patients were randomly assigned to the esketamine group (ES group) and dezocine group (DE group). In the ES group, 1.5 mg/kg esketamine was used. In the DE group, 0.3 mg/kg dezocine was used. Primary outcome measures were Visual Analog Scale (VAS) score at 4 h, 8 h, 24 h and 48 h after surgery. The second outcome measures were Interleukin-6 (IL-6) and C-reactive protein (CRP) levels in the serum 10 minutes before anesthesia induction, and at 24 h and 48 h after surgery.

**Results:**

The VAS scores at 4 h, 8 h, 24 h and 48 h after the surgery in the ES group vs DE group were 2.70 vs 3.50(*P*=0.013),2.35 vs 3.15(*P*=0.004),1.69 vs 2.58(*P*=0.002), and 1.50 vs 2.26(*P*=0.002), respectively. The serum IL-6 concentrations 10 minutes before anesthesia induction, and at 24 h and 48 h after surgery in the ES group and DE group were 34.39 and 34.12(*P*=0.901),112.33 and 129.60(*P*=0.014), and 89.69 and 108.46(*P*<0.001), respectively. The CRP levels in serum 10 minutes before anesthesia induction, and at 24 h and 48 h after the surgery in the ES group and DE group were 5.99 and 5.86(*P*=0.639), 28.80 and 35.37(*P*<0.001), and 23.17 and 30.11(*P*<0.001), respectively.

**Conclusion:**

For postoperative pain after LC, 1.5mg/kg esketamine provided better analgesia and reduced inflammation levels than 0.3mg/kg dezocine.

**Trial registration:**

This trial was registered in the China Clinical Research Information Center in 31/05/2023 : https://www.chictr.org.cn/bin/home (Registration number: ChiCTR2300072011).

## Background

Acute cholecystitis, which is caused by gallstones, is a common clinical disease. Despite diverse treatment methods, laparoscopic cholecystectomy (LC) remains the gold standard for the surgical treatment of calculous cholecystitis [1]. Compared to traditional open cholecystectomy, LC is a minimally invasive surgical procedure that offers patients smaller incisions and reduced trauma. It is still traumatic to the body as some patients experience severe pain after surgery. The pain sources from the abdominal wall and internal organs, and the insufflation of carbon dioxide during the procedure can result in shoulder pain due to diaphragmatic traction and stretching [2]. Postoperative pain leads to complications such as increased heart rate, increased blood pressure, anxiety, sleep disorders, activation and release of inflammatory factors, inhibition of the immune system, and restriction of early postoperative activities and eating, causing lower perioperative patient comfortability and lower patient satisfaction, as well as increased duration of hospital stays and medical costs [3].

Patient-controlled intravenous analgesia(PCIA) has always been a hot topic of concern for anesthesiologists. As one of the most powerful opioid analgesics currently available, sufentanil is widely applied to PCIA due to its rapid peak and short half-life [4]. However, it still shares the common shortcomings of opioids, such as respiratory depression, nausea, vomiting, bradycardia, fainting, low blood pressure and astriction, restricting its application in clinical practice alone [5]. Therefore, sufentanil is usually used clinically in combination with one or more other analgesics to meet the analgesic needs of patients while reducing adverse reactions to all kinds of drugs.

Current studies regard dezocine as a partial agonist μ receptor and κ receptor, which has analgesic and sedative effects by inhibiting the reabsorption of norepinephrine and serotonin, and can reduce sufentanil-mediated hyperalgesia by restricting calmodulin-dependent protein kinase II (CaMKII.) [6, 7]. The combination of dezocine with other μ-receptor agonists significantly enhances the analgesic effect of the drug. Studies have indicated that the combination of dezocine and sufentanil can effectively relief postoperative pain in patients undergoing laparoscopic cholecystectomy [8]. However, this approach still involves the combination of two opioid drugs, which significantly increases the dosage of opioids administered to patients and still has an impact on patient prognosis.

As a new drug applied for clinical trials in recent years, esketamine is an N-methyl-D-aspartate (NMDA) receptor blocker and a dextroisomer of ketamine, which has twice the affinity with NMDA receptors as ketamine. Notably, esketamine has a variety of advantages over ketamine, including high bioavailability, rapid onset and metabolism, minimal side effects and others [9]. Esketamine has been successfully applied in clinical fields such as cancer pain control, antidepressant therapy and perioperative analgesia. It has become a frontline medication in anesthesia due to its widespread use and effectiveness.

Scientists both domestically and internationally have employed the combination of esketamine and sufentanil as a method of analgesia, which has advantages such as strong analgesic effects, antidepressant properties, and minimal side effects. However, due to a lack of clinical evidence, the effectiveness of this regimen for pain management in patients with LC has still not been fully established [10]. Therefore, we explored the effects of esketamine combined with sufentanil for postoperative pain management in LC patients and evaluated its impact on postoperative inflammatory factors by comparing it with the combination of dezocine and sufentanil as a PCIA regimen to provide a novel PCIA approach.

## Methods

### Participants

This study was approved by the Ethics Committee of Zigong Hospital of Traditional Chinese Medicine (2022 Quick Approval NO.21) and all participating patients signed an informed consent form. Patients who were scheduled to undergo LC in Zigong Hospital of Traditional Chinese Medicine from June 2023 to August 2023 were selected. This trial was registered at the China Clinical Research Information Center (Registration number: ChiCTR2300072011).

The inclusion criteria were as follows: (1) willing to participate in this study; (2) conformed to ASA Classification I-II; (3) 18 years old ≤ age ≤ 65 years old; and (4) 18.5 kg/m^2^ ≤ BMI ≤ 24 kg/m^2^. The exclusion criteria were as follows: (1) had mental diseases; (2) had a history of substance abuse; (3) had contraindications to esketamine; (4) had allergies to narcotics. Quit standards were as follows: (1) refused to continue the experiment during the clinical trials; (2) had severe complications before and after the surgery, such as massive hemorrhage, infection, heart attack, respiratory failure, severe nausea and vomiting, and delirium; (3) resorted to open cholecystectomy during the surgery; (4) had a duration of surgery greater than 2 hours.

### Randomization and blinding

The 58 included patients were numbered 1-58 sequentially, and the 58 random numbers corresponding to these 58 patients were generated by the random number generator of the SPSS software (version 27.0; IBM Corporation, Armonk, USA). A total of 58 random numbers were randomly assigned to the ES group or DE group at a 1:1 ratio. Before the surgery, an anesthesia nurse handed the configured analgesic pumps to the anesthesiologist in numbered order. Furthermore, physicians involved in surgical anesthesia and postoperative visits were unaware of the grouping details. The grouping of patients will be announced only if necessary, and the sample will quit the study group at the same time.

### Intervention

In the ES group, 2 µg/kg sufentanil, 1.5 mg/kg esketamine, and 0.2 mg/kg ondansetron were used for PCIA, which was diluted to 100 ml in total with normal saline. The initial dose was 2 ml, with a continuous infusion of 2 ml/h, a bolus dose of 0.5 ml, and a locking duration of 15 minutes [11]. In the DE group, PCIA was administered 2 µg//kg sufentanil, 0.3 mg/kg dezocine, or 0.2 mg/kg ondansetron, while the other parameters remained the same as those in the ES group [12].

### Anesthetic management

Patients were informed of the usage of the analgesic pump in detail one day before surgery, and were told of the usage of the VAS score until they were proficient in using it. Patients were also advised to get out of bed as soon as possible if the postoperative pain was tolerable.

When patients entered the operating room, noninvasive blood pressure, oxygen saturation, ECG, PETCO_2_, muscle relaxation monitoring-TOF, and depth monitoring-BIS were monitored until the patients left the operation room. All preoperative drugs were administered before induction. Anesthesia induction: 0.05 mg/kg midazolam, 0.3-0.5 µg/kg sufentanil, 0.15 mg/kg cisatracurium, and 1.5-2 mg/kg propofol. Anesthesia maintenance: Endotracheal intubation was performed when the BIS values fell between 40 and 50 remained stable for 1 minute and when the TOF was ≤ 0. If anesthesia induction and anesthesia maintenance went smoothly, the anesthesia machine was linked and volume-controlled ventilation was carried out. The parameters of the anesthesia machine were set as follows: tidal volume 6-8 ml/kg, respiratory rate 10-15 times/min, PEEP 4-8 mmHg, and oxygen concentration 30%-50%. The parameters were dynamically adjusted, adopting the minimum value on the basis of ensuring PETCO_2_ ≤ 45 mmHg and airway pressure < 30 cm H_2_O, SPO_2_ > 90%. The BIS was maintained at 40-60 with 1.5%-3% sufentanil and 0.05µg-0.2 µg/kg/min remifentanil. Cisatracurium was given after TOF ≥ 2 with intermittent push to 1/3 of the induction dose, and the induction dose was removed 30 minutes before the end of the operation. Muscle relaxants were removed 30 minutes before the end of surgery. The sevoflurane volatilization jar was closed when suturing the skin, and the sufentanil infusion was removed at the end of the surgery. After the surgery, 4 mg of ondansetron was given to prevent vomiting. Then the analgesic pump was linked and 0.04 mg/kg neostigmine plus 0.02 mg/kg atropine was given to antagonize muscle relaxation after TOF ≥ 2. When the BIS >70, the patient needed to be awake until the eyes were opened. Those who met the indications for an awake withdrawal catheter were removed from the tracheal tube and sent to the recovery room. If the patient’s VAS score was >5 and PCA was ineffective, an intravenous drip of 40 mg parecoxib was given.

### Outcome measures

The Primary Outcome Measures included the VAS score at 4 h (T1), 8 h (T2), 24 h (T3) and 48 h (T4) after arriving at postanesthesia care unit (PACU) to assess the pain of patients. The VAS score ranges from 0 points to 10 points, with 0 points indicating no pain and 10 points indicating unbearable severe pain. The higher the score, the more unbearable the pain.

The Secondary Outcome Measures:(1)the number of analgesic pump presses and the dosage of parecoxib within 48 hours after the surgery; (2)the dosage of sufentanil and remifentanil used during the surgery;(3)the initial bedside activity time;(4)assessment of sleep quality index of patients one month before surgery, one day and two days after the surgery by Athens insomnia scale(AIS). Each item is graded into 4 levels, with 3 = severe, 2=significant, 1=slight and 0=no problem/normal. The total score is the sum of all items, with a lowest score of 0 and a highest score of 21. A total score<4 indicates no sleep disorder; 4≤total score≤6 indicates suspected insomnia; and a total score>6 indicates insomnia;(5)Postoperative adverse reactions: No adverse reactions, nausea and vomiting, faint and headache, itchy skin, respiratory depression, dream-disturbed sleep and nightmare, diplopia, hallucination and delirium;(6)Assessment of Interleukin-6 (IL-6) and C-reactive protein (CRP) levels in serum: 3 ml of peripheral venous blood was drawn from patients 10 minutes before anesthesia induction, 24 hours and 48 hours after the surgery, and IL-6 and CRP levels in serum were measured by immune nephelometry.

### Statistical analysis and sample size

According to previous clinical data,and our preliminary experiment, the VAS scores at 4h after surgery were 2.55 and 3.21 in ES group and DE group respectively. α=0.05 and β=0.2 were selected for the two-sided test and a 1-point deduction of VAS was defined to be more effective. The needed sample size of each group was 24 cases, which was calculated by applying the PASS software(version 11.0; NCSS Corporation, Utah, USA) . The expected loss to follow-up reached 20%, so the final decision was made to include 58 patients.

The SPSS software(version 27.0; IBM Corporation, Armonk, USA) was adopted to carry out data analysis and data processing. The quantitative data were tested for normality first. If the data of two groups conformed to a normal distribution or approximately (such as VAS score, inflammatory factors, and AIS score), then the mean ± standard deviation was expressed, a t-test was selected for comparison between groups and analysis on variance of repeated measurement designed data was used for comparison within groups. If not (such as the number of analgesic supplements), the median was expressed (the 1^st^ quartile and 3^rd^ quartile) and the Mann-Whitney U test was selected. The counting data (such as sex and adverse reactions) were expressed by frequency and (or) percentage (%), and the chi-square test was used for comparison between groups. *P*<0.05 was considered statistically significant. All data were reserved up to two decimal places.

## Results

**Fig. 1:**
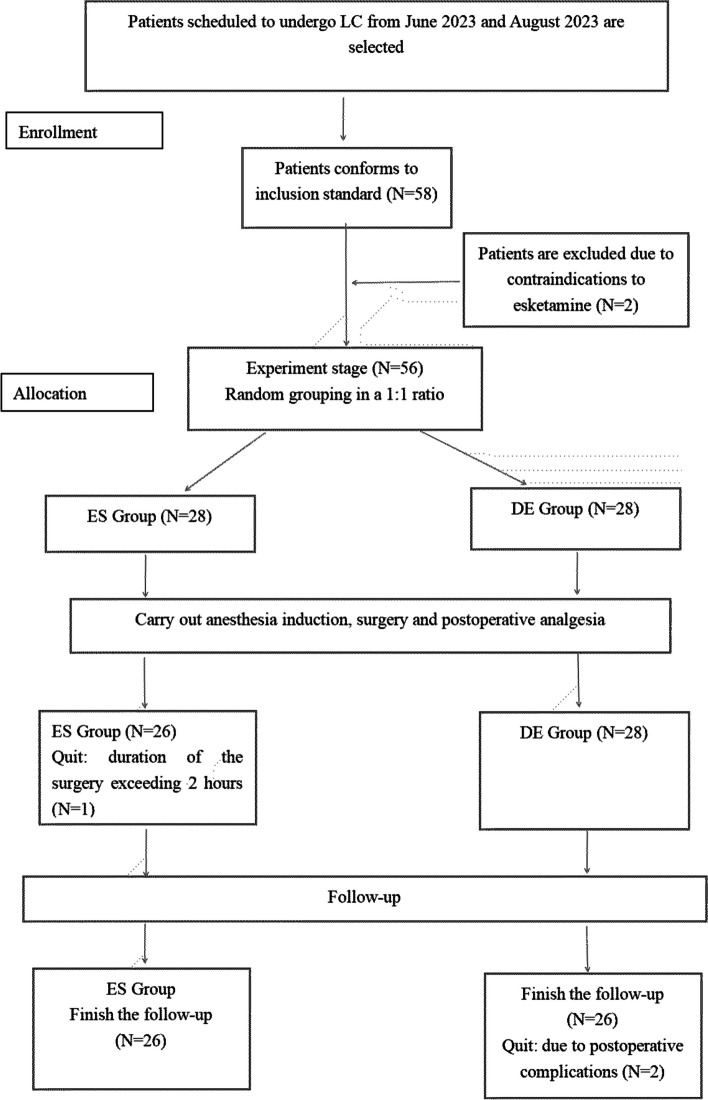
Sample Screening Process Based on Unified Standard. ES group-esketamine group,DE group-dezocine group

In total, this study recruited 58 patients who underwent laparoscopic cholecystectomy under general anesthesia, of which 2 patients were excluded due to contraindications to esketamine, 1 patient quit the study due to the duration of the surgery exceeding 2 hours, 1 patient quit due to a change to open cholecystectomy during the surgery and 2 patients quit due to complications(infection and bleeding). Finally, 52 patients completed this study (ES group, *n*=26; DE group, *n*=26) (Fig. 1).

The baseline features of patients in the two groups, including age, sex, BMI, duration of the surgery, and ASA classification, were not significantly different (*P*>0.05) (Table 1).

**Table 1:** Patients’ baseline features

	Age(Years)	Sex (Male/Female)	BMI(kg/m^2^)	Surgery Duration	ASA(I/II)
ES Group (*N*=26)	44.15±10.36	11/15	22.32±1.53	48.00±8.26	16/10
DE Group (*N*=26)	43.19±10.44	9/17	21.83±1.45	48.54±8.96	18/8
*P* Value	0.740	0.569	0.236	0.823	0.560

Age, BMI and Surgery Duration are expressed as the mean ± standard deviation while Sex and ASA are expressed as frequency

*BMI* Body Mass Index, *ASA* American Society of Anesthesiologists, *ES group* Esketamine group, DE group-dezocine group

### General condition of postoperative pain

**Fig. 2:**
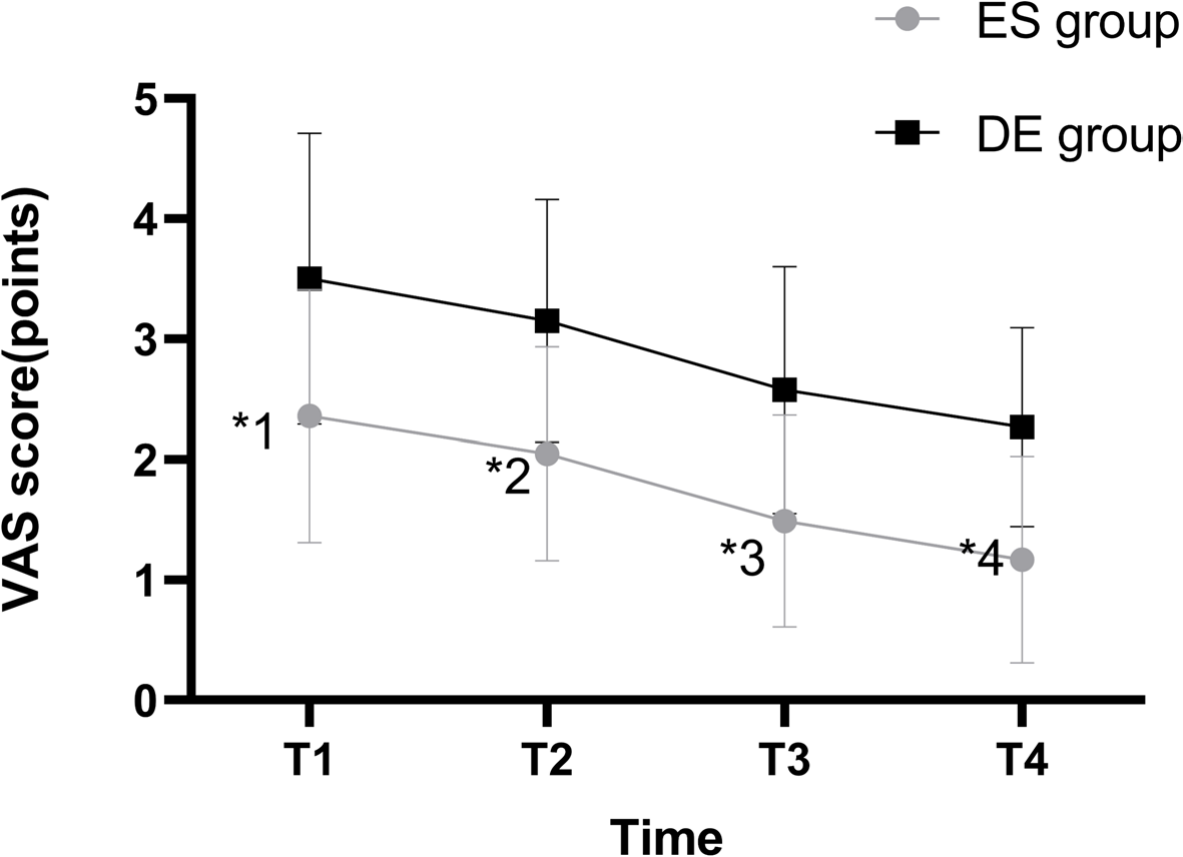
Comparison and Variation Trends of VAS Scores of the Two Groups. T1-4 hours after the LC; T2-8 hours after the LC; T3-24 hours after the LC; T4-48 hours after the LC,ES group-esketamine group,DE group-dezocine group. In comparison with the DE Group, ^*1^*P*=0.013,^*2^*P*=0.004,^*3^*P*=0.002,^*4^*P*=0.002. VAS, Visual Analogue Scale

The VAS scores of the ES group at four time points, T1, T2, T3, and T4 were all lower than those of DE group (*P*<0.05), and differences between patients in each group at T1, T2, T3,and T4 were statistically significant (*P*<0.05) (Fig. 2). There was no statistically significant difference (*P*>0.05) in the dosage of sufentanil and remifentanil between the two groups of patients. The number of analgesic pump presses and initial bedside activity time of the ES group were lower than those of the DE group (*P*<0.05), and the dosage of analgesic supplements of parecoxib in the ES group was lower than that in the DE group (*P*<0.05) (Table 2).

**Table 2:** Evaluation of analgesic effect

	ES Group(*N*=26)	DE Group(*N*=26)	*P* Value
The dosage of sufentanil(μg)	21.63±3.25	21.58±3.52	0.961
The dosage of remifentanil(μg)	252.40(226.48, 281.10)	259.80(216.00, 299.90)	0.949
The number of analgesic pump presses(Times)	2.85±1.57	4.89±2.49	<0.001
The dosage of parecoxib (mg)	0(0,40)	40(0,80)	0.0245
The initial bedside activity time (h)	5.46±1.79	6.65±1.79	0.020

The dosage of sufentanil,the number of analgesic pump presses and the initial bedside activity time are expressed as the mean ± standard deviation ;The dosage of remifentanil and the dosage of parecoxib are expressed as the median (the 1^st^ quartile and 3^rd^ quartile).

*ES group* Esketamine group, *DE group* Dezocine group

### Sleep quality

**Fig. 3:**
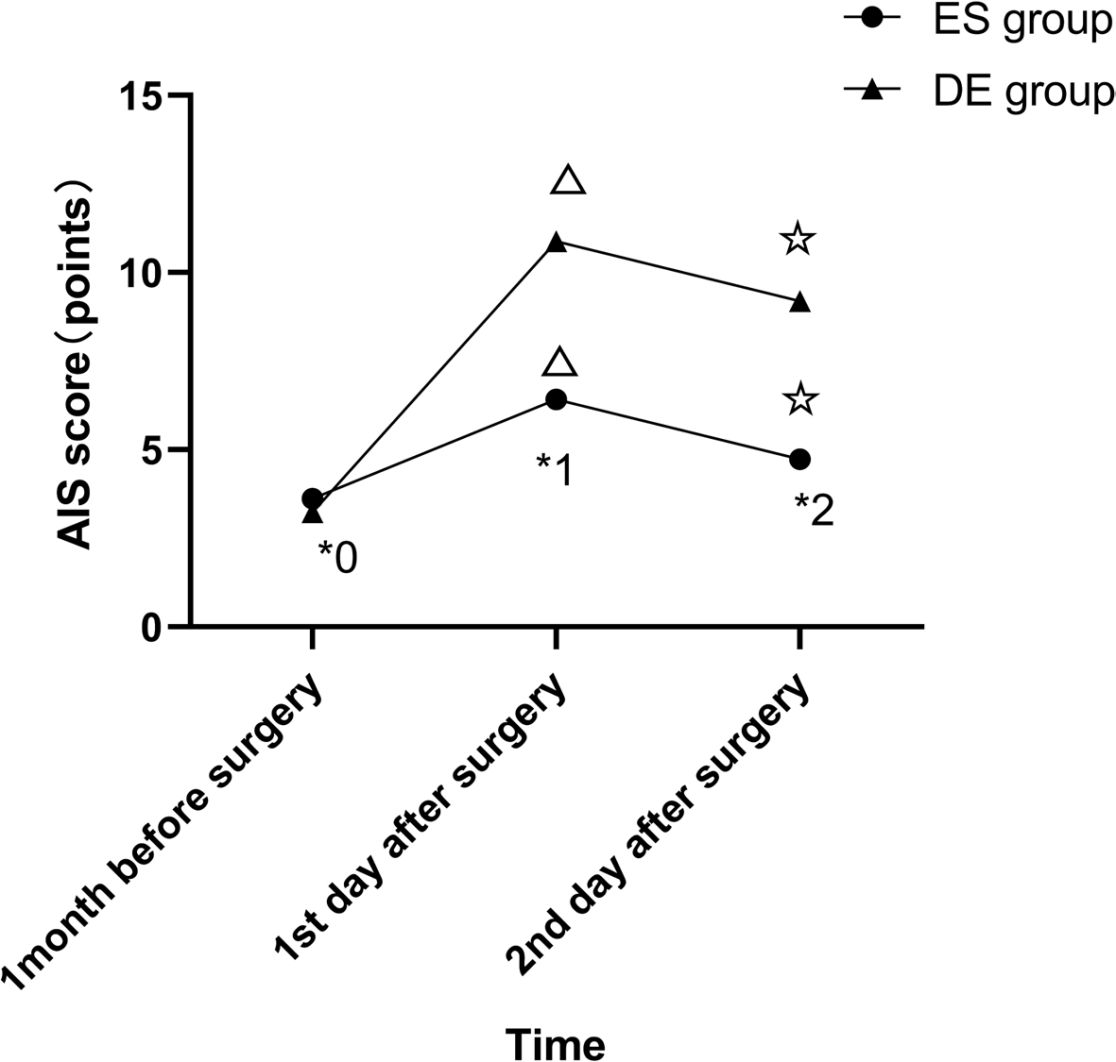
Comparison of AIS Scores at Different Time Points of the Two Groups. ^*0^*P*=0.416, ^*1^*P*<0.001,^*2^*P*<0.001, in comparison with the DE group; ^△^*P*<0.001, in comparison with 1 month before the surgery; ^☆^*P*<0.001, in comparison with 1 day before the surgery. AIS-Athens Insomnia Scale,ES group-esketamine group,DE group-dezocine group

The AIS scores of the patients in the two groups one month before the surgery were not significantly different (*P*>0.05). The AIS scores at one day and two days postoperatively were significantly lower in the ES group than in the DE group (*P*<0.05); within each group, the scores were higher at one day postoperatively than at one month preoperatively (*P*<0.05) and lower at two days postoperatively than at one day postoperatively (*P*<0.05)( Fig. 3).

### Adverse reactions

The main adverse reactions observed in both groups of patients were nausea and vomiting. However, the difference in the incidence between the two groups was not statistically significant (*P*>0.05)(Table 3).

**Table 3:** Adverse reactions

	PONV [Case (%)]	Faint or headache [Case (%)]	Itchy skin [Case (%)]	Dream-disturbed sleep or nightmare [Case (%)]	Diplopia [Case (%)]	Hallucination [Case (%)]
ES Group (*N*=26)	4(15.38)^*^	2(7.69)^*^	0(0)^*^	1(3.85)^*^	1(3.85)^*^	1(3.85)^*^
DE Group (*N*=26)	7(26.92)	0(0)	2(7.69)	2(7.69)	0(0)	0(0)
*P* Value	0.308	0.490	0.490	1.000	1.000	1.000

Categorical variables are expressed as numbers (proportions)

*PONV* Postoperative Nausea and Vomiting, *ES group* Esketamine group, *DE group* Dezocine group

### Inflammatory factors

**Fig. 4:**
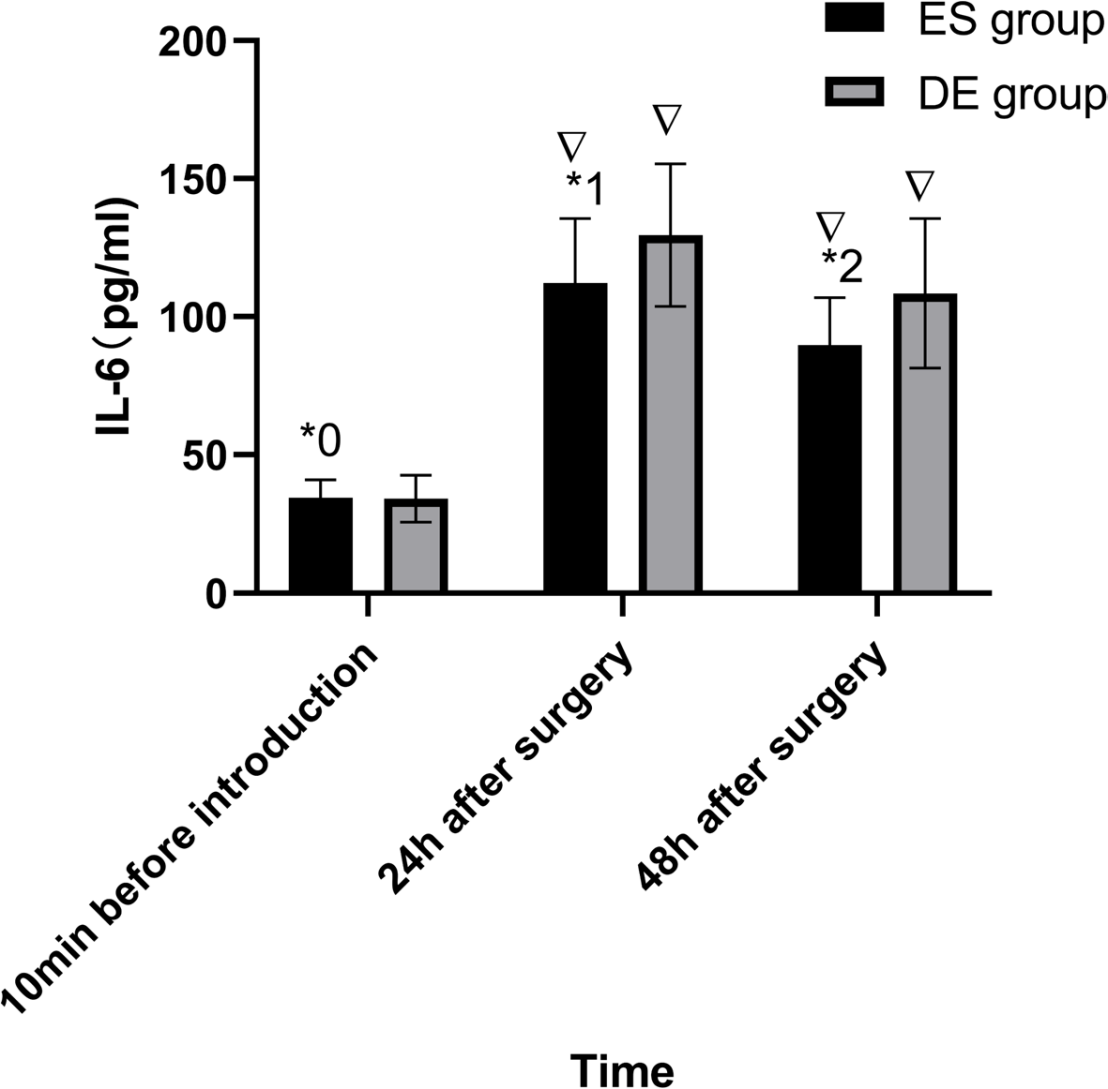
Comparison of IL-6 Level of the Two Groups. ^*0^*P*=0.901, ^*1^*P*=0.014,^*2^*P*=0.004,in comparison with the DE group; ^▽^*P*<0.001, in comparison with 10 minutes before anesthesia induction within groups. IL-6, Interleukin-6; pg/ml, picograms per milliliter; ES group, esketamine group;DE group, dezocine group;

**Fig. 5:**
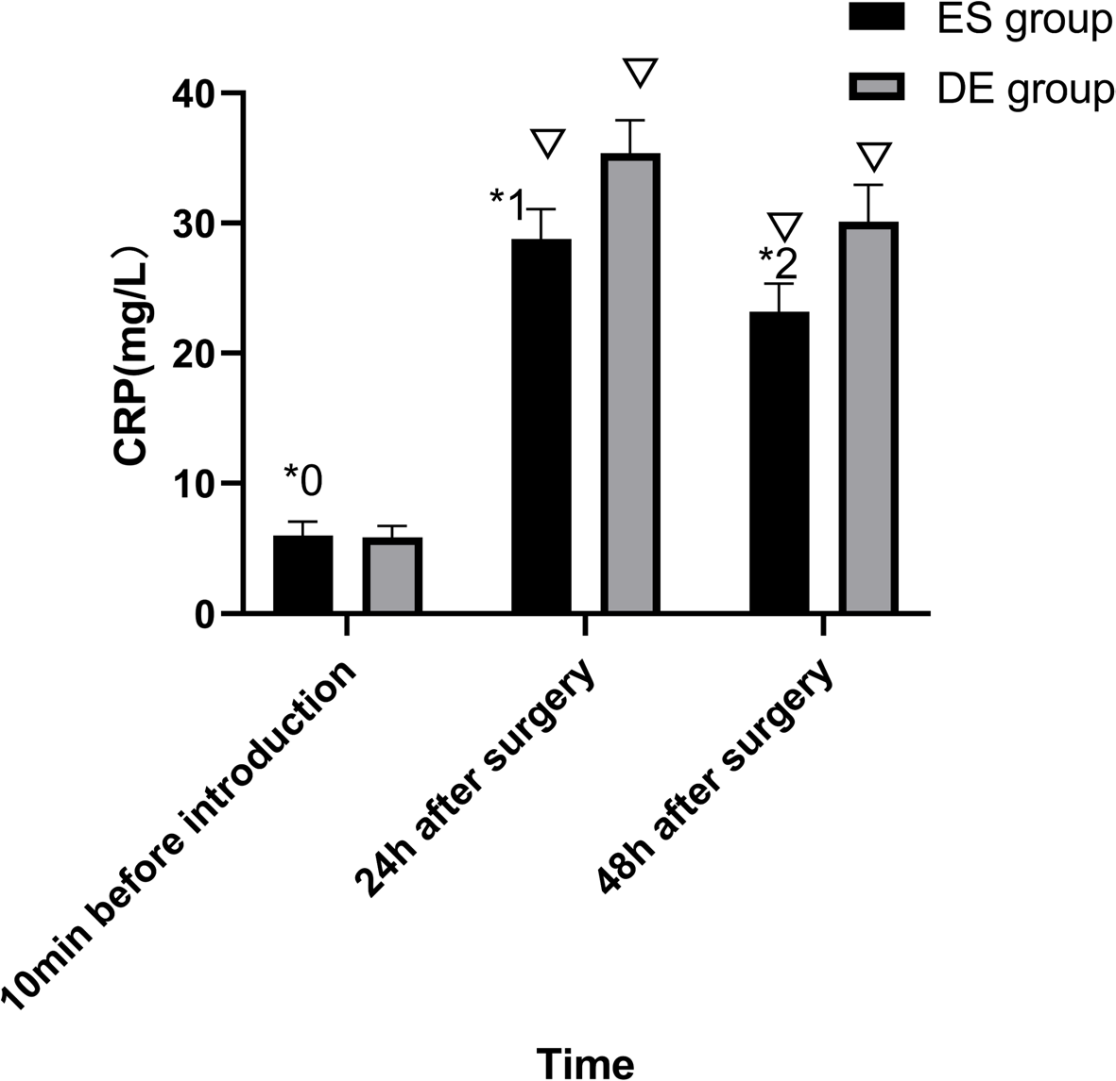
Comparison of CRP Level of the Two Groups. ^*0^*P*=0.639, ^*1^*P*<0.001,^*2^*P*<0.001,in comparison with the DE group; ^▽^*P*<0.001, in comparison with 10 minutes before anesthesia induction within groups. CRP, C-reactive Protein; mg/L,milligrams per liter; ES group, esketamine group; DE group, dezocine group

The IL-6 and CRP levels of patients in the two groups 10 minutes before anesthesia induction were not significantly different (*P*>0.05). The IL-6 and CRP levels of patients in the ES group at 24 hours and 48 hours after the surgery were lower than those of the DE group (*P*<0.05). The differences between the results of patients in each group at 24 hours and 48 hours after the surgery and 10 minutes before anesthesia induction were statistically significant (*P*<0.05) (Figs. 4 and 5).

## Discussion

This study compared the esketamine and dezocine in combination with sufentanil to prevent the inflammatory pain after LC. We believe that 1.5 mg/kg esketamine in combination with sufentanil is better than 0.3 mg/kg dezocine in combination with sufentanil in terms of analgesic effects, improvement of postoperative sleep quality and reduction of inflammatory factor release, without increasing the incidence of adverse reactions.

The best time to manage postoperative pain is within 48 hours after surgery, especially within the first few hours. Considering the timeliness of the postoperative interview, we excluded patients whose data might have involved late at night or early in the morning. The VAS score is often used to evaluate the degree of acute postoperative pain in clinical practice. Based on the conclusions of previous studies, we defined a 1-point difference in VAS score as clinically meaningful [13]. Both PCIA plans showed excellent analgesic effects. We discovered that VAS scores at T1, T2, T3 and T4 after surgery were significantly lower in the ES group than in the DE group, and the number of analgesic pump presses, the number of analgesic supplements taken within 48 hours after the surgery and the initial bedside activity time in the ES group were lower than those in the DE group. This demonstrated that esketamine in combination with sufentanil has a better analgesic effect at the set dose. Zhu et al. suggested that dezocine can reduce postoperative pain to some extent by enhancing the analgesic effect of sufentanil [14]. Esketamine has demonstrated a notable superiority over dezocine in effectively reducing postoperative pain caused by laparoscopic surgery. As previous research noted, continuous infusions of low doses of esketamine infusion were effective in reducing postoperative pain [15]. Similarly, the administration of subanesthetic doses of esketamine during laparoscopic total hysterectomy has been shown to improve the treatment of postoperative depression and pain alleviation [16]. These findings further support our result that esketamine possesses potent analgesic effects after laparoscopic surgery. The powerful analgesic effect of esketaminem may be due to its prolonged antagonistic effect on NMDA receptors and the fact that its active metabolites also have an analgesic effect [17]. Notably, VAS score was greater in the ES group in our study than in previous reports at various time points. This discrepancy may be attributed to the higher esketamine infusion rate (0.3 mg/kg/h) used during the surgery, which significantly exceeded the postoperative infusion rate in our study. Despite these findings, patients expressed high overall satisfaction with the treatment protocol, indicating its clinical feasibility.

Postoperative sleep disorder (PSD) is related to postoperative delirium, cognitive dysfunction, pain and even cardiovascular accidents in severe cases, which significantly delay postoperative recovery [18]. The reasons are surgical stress response, pain, general anesthesia, and the usage of opioids. Therefore, improving postoperative sleep quality is an essential parameter that anesthesiologists need to consider. In this study, the PCIA regimen in the ES group demonstrated greater effectiveness in improving postoperative sleep quality, which indicated that esketamine, compared to dezocine, can more effectively enhance patients’ postoperative sleep quality and increase overall satisfaction. Similar to our observations, the results of Qiu et al. also concluded that esketamine can prevent PSD [19]. In this study, we found that the lower VAS scores in the ES group were associated with PSD, indicating that the effect of esketamine in improving sleep disorders is related to its ability to reduce postoperative pain.

Surgical trauma can induce various physiological stress responses, and surgical pain is closely associated with inflammatory factors. Several studies have shown that postoperative pain is primarily attributed to inflammatory factors. When the body is injured, it releases various inflammatory factors, with IL-6 and CRP being notable epresentative factors, which were commonly observed in surgeries such as cholecystectomy, appendectomy, and colon resection [20–23]. The levels of inflammatory factors are related to the extent of surgical trauma, and the durations at which different inflammatory factors reach their peak are different. The peak times of IL-6 and CRP were 12-24h and 24-48h after LC, respectively [24]. Additionally, the inflammatory response triggered by trauma not only contributes to postoperative pain but exacerbates perioperative inflammation and immune suppression [25]. Therefore, postoperative administration of esketamine, as a potent analgesic, can effectively reduce both postoperative pain and inflammatory factor levels. Tu et al.’s study revealed that the application of esketamine during anesthesia induction for the elderly improved the postoperative inflammatory response [26]. To further investigate the analgesic effects of esketamine and the underlying causes of pain, we measured the levels of IL-6 and CRP in patients at 24 hours and 48 hours postoperatively. In this study, we found that IL-6 and CRP levels in the ES group at 24 hours and 48 hours postoperatively were lower than those in the DE group, suggesting that esketamine is capable of reducing inflammatory cytokine levels and that esketamine in combination with sufentanil is more effective in reducing the level of inflammatory factors at the set dose. In our current study, we found a consistent association between lower levels of IL-6 and CRP and lower VAS scores in postoperative patients. These findings indicated that postoperative pain is influenced by inflammatory factors, and that esketamine may reduce pain levels by mitigating the inflammatory response. Therefore, our study provides support for the idea that esketamine can effectively alleviate inflammatory pain.

Research has indicated that the combination of esketamine and dezocine, respectively, with sufentanil in postoperative PCIA can enhance the analgesic effect while reducing adverse reactions, thereby allowing for a reduction in sufentanil dosage [27, 28]. Compared to dezocine, the combination of esketamine and sufentanil has been found to effectively decrease the adverse effects associated with opioids. However, esketamine itself can produce certain side effects, such as vomiting, dizziness, and headaches, which are dose-dependent [29]. To minimize these adverse reactions, we opted for an esketamine dosage of 1.5 mg/kg (0.03 mg/kg/h). Nevertheless, considering the possibility of increased nausea and vomiting following esketamine administration, we closely monitored and observed the patients for adverse reactions. In our study, after administering esketamine at a dosage of 1.5 mg/kg (0.03 mg/kg/h), we found that the incidence of nausea and vomiting in this group was not higher than that in the control group, and there were no severe cases reported, suggesting the superiority of esketamine over dezocine.

Some limitations are obvious in this study. First, we applied scales to assess the intensity of pain and sleep quality, which was largely restricted by the subjective awareness of patients and follow-up staff, leading to potential bias in results. Second, it is unknown whether the dose of esketamine in this study is the optimal dose, and it needs to be further explored. And whether a dose of 1.5mg/kg is equivalent to dezocine at 0.3mg/kg is also unclear.The occurrence rate of postoperative nausea and vomiting and other adverse reactions remains high, and it may be necessary to consider implementing new experimental methods to further improve the management of postoperative nausea and vomiting. Finally, our observation period is limited to 48 hours, and the clinical significance of the observed effects over a longer duration is yet to be explored. Despite the widespread acceptance of esketamine in clinical practice, there are still many fields that require further investigation and exploration, necessitating the use of larger sample sizes and diverse research methodologies.

## Conclusion

The combined administration of 1.5 mg/kg esketamine and sufentanil for postoperative PCIA in patients undergoing LC yielded significant improvements in pain relief and a reduction in inflammatory cytokine levels. Esketamine can reduce the expression levels of inflammatory factors, which is associated with alleviating inflammatory pain.

## Data Availability

The data used to support the findings of this study are available from the corresponding author upon request.

## Abbreviations

LC: Laparoscopic Cholecystectomy
ES: Esketamine
DE: Dezocine
VAS: Visual Analog Scale
IL-6: Interleukin-6
CRP: C-reactive protein
PCIA: Patient Controlled Intravenous Analgesia
NMDA: N-methyl-D-aspartate
BMI: Body Mass Index
ECG: Electrocardiograph
PetCO_2_: End-tidal Carbon Dioxide Partial Pressure
TOF: Train of Four Stimulation
PEEP: Positive End Expiratory Pressure
SPO_2_: Degree of Blood Oxygen Saturation
PACU: Postanesthesia Care Unit
AIS: Athens Insomnia Scale
PSD: Postoperative Sleep Disorder
